# White Sponge Nevus: A Case Report

**DOI:** 10.5681/joddd.2009.017

**Published:** 2009-06-05

**Authors:** Amirala Aghbali, Firouz Pouralibaba, Hossein Eslami, Farzaneh Pakdel, Zahra Jamali

**Affiliations:** ^1^Assistant Professor, Department of Oral and Maxillofacial Pathology, Faculty of Dentistry, Tabriz University of Medical Sciences, Tabriz, Iran; ^2^Assistant Professor, Department of Oral Medicine, Faculty of Dentistry, Tabriz University of Medical Sciences, Tabriz, Iran; ^3^Post-graduate Student, Department of Oral Medicine, Faculty of Dentistry, Tabriz University of Medical Sciences, Tabriz, Iran

**Keywords:** Dyskeratosis, white lesion, white sponge nevus

## Abstract

White sponge nevus (WSN) is a rare hereditary dyskeratotic hyperplasia of mucous membranes. It is an autosomal dominant disorder with variable penetrance. We report a case of WSN in a healthy 21-year-old male with no history of familial involvement. A white smooth plaque with no erythema or other structural abnormalities was observed, which confirmed the diagnosis of WSN histopathologically.

## Introduction

White sponge nevus (WSN) is a relatively rare cutaneous and mucosal lesion.^1,2,3^ Hyde reported the first case of WSN in 1909 and a detailed report was published in 1935 by Cannon.^4,5^ Etiologically, it is a rare developmental anomaly inherited as an autosomal dominant trait with variable expressivity and a high degree of penetrance. This condition is attributed to a defect in the normal keratinization (keratin 4 and keratin 13, which are specifically expressed in the spinous cell layer of the oral mucosa). ^6 - 11^ This keratotic mucosal alteration may be seen on vaginal and rectal mucosa but the great majority of cases involve the oral mucosa.^1^

A search of dermatological and gynecological literature revealed very little about WSN in Iran. More information was available about this lesion in the oral cavity, which was retrieved from the dermatological and dental literature.^12^

Lesions of WSN usually appear at birth or in early childhood, but sometimes the condition develops during adolescence. The lesions consist of symmetric, thickened, white, corrugated or velvety, diffuse plaques. Buccal mucosa is the most frequently affected, followed by the labial and gingival mucosa, and the floor of the mouth. Extra-oral mucosal sites, such as the nasal, esophageal, laryngeal, and anogenital mucosa, appear to be less commonly affected. Patients are usually asymptomatic. The white color does not diminish when the tissue is stretched in any mucosal site. ^1,2,6 ,13,14^

The recognition of this disorder is important in that it must be differentiated from other congenital or familial disorders of more widespread clinical significance. The clinical appearance is so distinctive that biopsy is usually unnecessary. The microscopic features of WSN are characteristic but not necessarily pathognomonic. Prominent hyperparakeratosis and marked acanthosis with clearing of the cytoplasm of the cells in the spinous layer are common features; however, similar microscopic findings may be associated with leukoedema and hereditary benign intraepithelial dyskeratosis. In some instances an eosinophilic condensation is noted in the perinuclear region of the cells in the superficial layers of the epithelium, a feature that is unique to WSN.^1-5^

In this paper, a case of WSN in a healthy white male with no history of familial involvement is described.

## Case Report

The patient was a 21-year-old Iranian male referred to the Department of Oral Medicine at Tabriz University of Medical Sciences Faculty of Dentistry for diagnosis and management of a “white, itchy spot” on the buccal mucosa. White bilateral lesion in oral mucosa was the chief compliant of the patient. The patient complained of a white lesion which was present since birth.

The patient’s general health was reportedly good. The patient denied presence of a similar condition in immediate family members or any similar lesions elsewhere on his body.

In clinical examination, there were bilateral, symmetrical white plaques and patches on the buccal and labial mucosa, which could not be removed (Figure 1). The plaques were smooth with velvety texture and irregular, well-defined borders. There was no elevation or erythema. The margins were clear and no lymph nodes were noticeable. Oral hygiene was good and other oral structures were normal in appearance.


Figure 1. Clinical views of the lesions.

**Figure:**
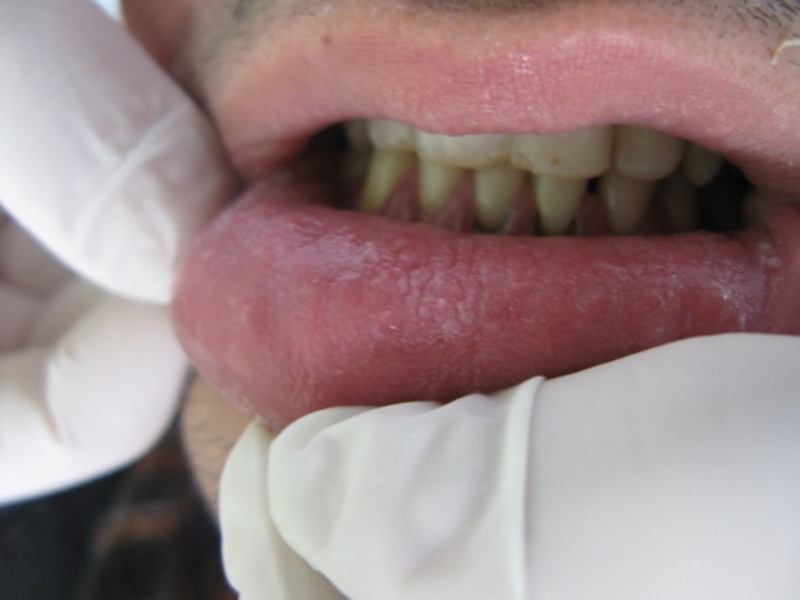

**Figure:**
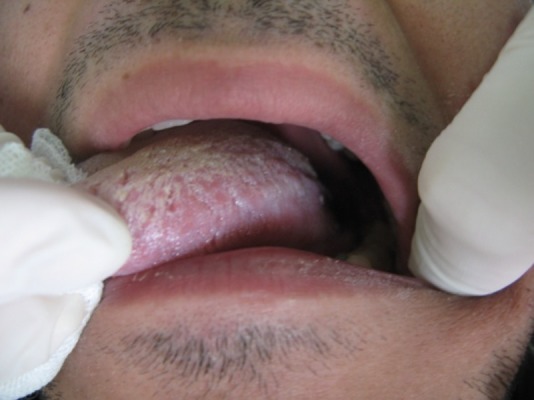

**Figure:**
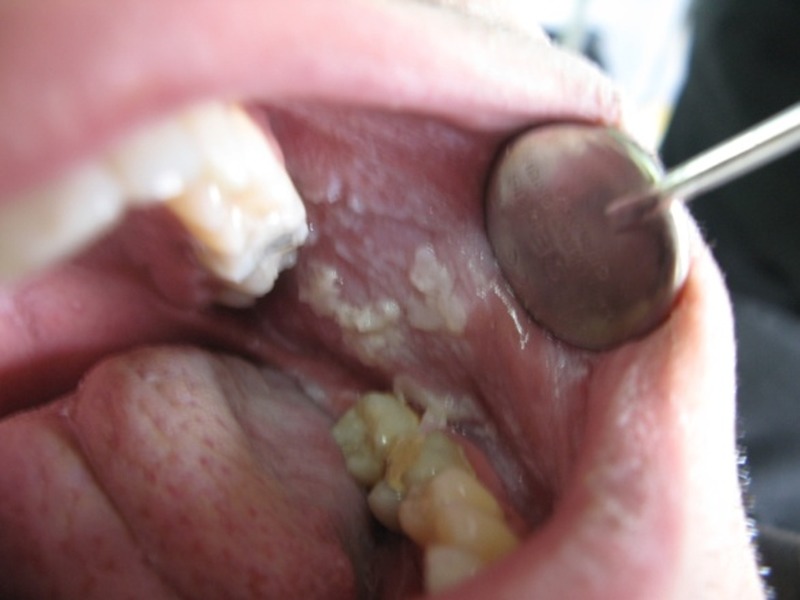

In histopathologic evaluation, oral mucosa covered by stratified squamous epithelium revealed prominent hyperparakeratosis and marked acanthosis with clearing of the cytoplasm of cells in the spinous layer. In addition, eosinophilic condensation was noted in the perinuclear region of the cells in superficial layers (Figure 2). Underlying connective tissue was normal in appearance with rare chronic inflammatory cell infiltration.


Figure 2. (a) Histopathologic view of the lesion (×10); (b) perinuclear condensation of keratin tonofilament (arrow) (×40) (H&E).

**a:**
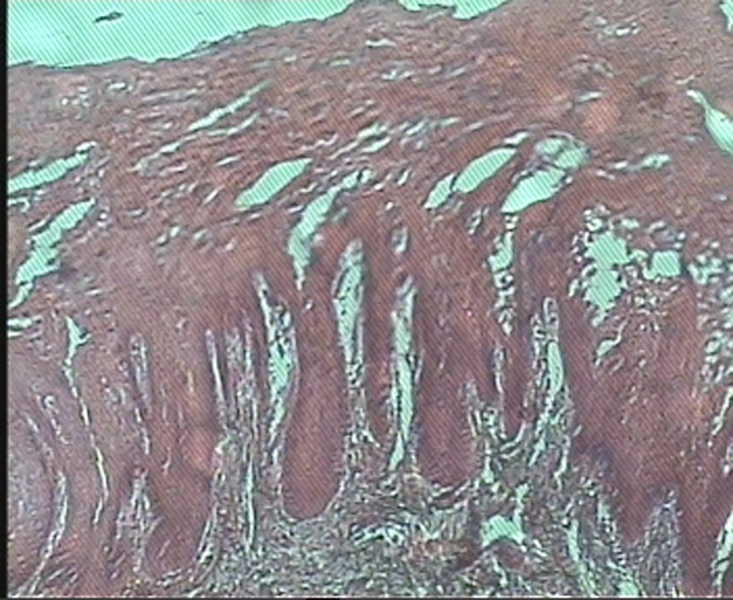

**b:**
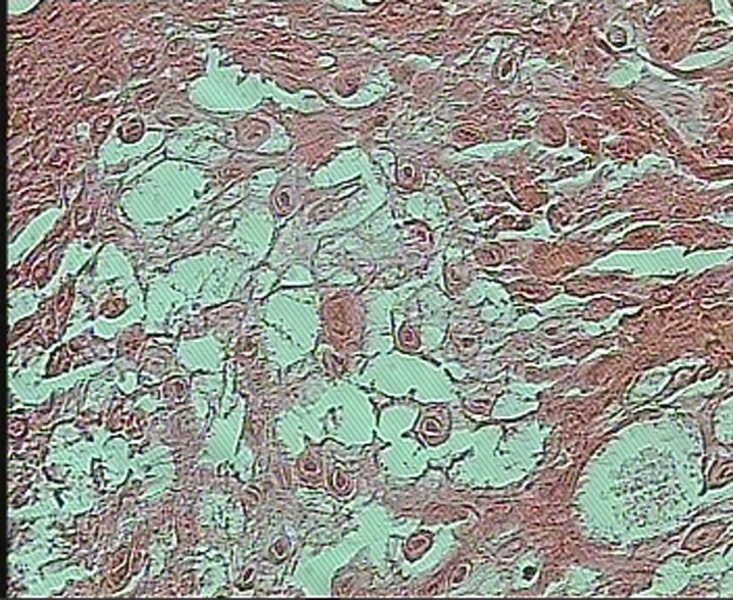

Based on clinical data and histopathologic findings, the lesion was consistent with white sponge nevus. Because of benign nature of this lesion, no treatment is necessary and only biopsy and correct diagnosis is necessary to rule out other similar lesions. Six-month follow-up was recommended.

## Discussion

WSN is a rare hereditary dyskeratotic hyperplasia of mucous membranes. This entity is also known by other names, such as Cannon's disease, familial white folded hypertrophy of the mucous membranes, hereditary leukokeratosis, white gingivostomatitis, and exfoliative leukoedema.^1-5^ WSN is an autosomal dominant disorder with variable penetrance and hence familial reports are not very common, similar to the present case. WSN has been listed as a rare disorder, with a prevalence rate below 1 in 200,000.^13^ Most commonly, lesions appear at birth or in early childhood. Neither gender nor racial predilection exists.^14^ A case of WSN, in which human papilloma virus type 16 was demonstrated, has been reported in the literature.^4^ Many different types of white lesions can occur in the oral mucosa and the appearance of WSN is not pathognomonic. There is a need for precise identification through prompt histopathologic examination to differentiate this condition from more serious, potentially premalignant lesions as well as other genodermatoses such as hereditary benign epithelial dyskeratosis, lichen planus, lichenoid drug reaction, lupus erythematosus, cheek chewing and possibly candidiasis. While some of these lesions are benign, others are pre-malignant or manifestations of some systemic diseases. Therefore, early diagnosis of this benign lesion is important,^3^ and often, these lesions need different treatment plans.^15-21^ In addition, these lesions reveal different epidemiological patterns and involve different societies and races. In Northwest Iran, this condition seems to be rare and no other similar documented cases are available. In this case, none of the family members had similar lesions. This lesion appeared early in life without any reported changes throughout the patient’s life, but diffuse spreading of the lesion seems to be an alarming factor. Biopsy in such cases is necessary for treatment planning and ruling out of other lesions.
